# Veterinarian perceptions and practices in prevention and control of influenza virus in the Midwest United States swine farms

**DOI:** 10.3389/fvets.2023.1089132

**Published:** 2023-02-03

**Authors:** Daniel C. A. Moraes, Amy L. Vincent Baker, Xin Wang, Zhengyuan Zhu, Emily Berg, Giovani Trevisan, Jianqiang Zhang, Swaminathan Jayaraman, Daniel C. L. Linhares, Phillip C. Gauger, Gustavo S. Silva

**Affiliations:** ^1^Department of Veterinary Diagnostic and Production Animal Medicine, Iowa State University, Ames, IA, United States; ^2^Virus and Prion Research Unit, National Animal Disease Center, USDA-ARS, Ames, IA, United States; ^3^Department of Statistics, Iowa State University, Ames, IA, United States

**Keywords:** IAV, survey, veterinary practices, zoonotic disease, swine

## Abstract

Influenza A virus (IAV) is an endemic respiratory pathogen affecting swine worldwide and is a public health concern as a zoonotic pathogen. Veterinarians may respond to IAV infection in swine with varied approaches depending on their perception of its economic impact on human and animal health. This study considered three primary veterinary practice categories: swine exclusive veterinary practitioner, large animal practitioner, which corresponds to veterinarians that work predominantly with food animals including but not exclusively porcine, and mixed animal practitioner, which corresponds to veterinarians working with companion and food animals. This survey aimed to assess U.S. veterinarian perceptions, biosecurity practices, and control methods for IAV in swine. In this study, 54.5% (188/345) of the veterinarians that were targeted responded to all portions of the survey. The study results presented different perceptions regarding IAV among veterinarians in different types of veterinary practices and the current IAV mitigation practices implemented in swine farms based on strategic decisions. Collectively, this study also revealed the veterinarians' perceptions that IAV as a health problem in swine is increasing, IAV has a moderate economic impact, and there is a high level of concern regarding IAV circulating in swine. These findings highlight the need for IAV surveillance data, improved vaccine strategies, as well as important opportunities regarding methods of control and biosecurity. Additionally, results of this survey suggest biosecurity practices associated with the veterinarian's swine operations and prevention of zoonotic diseases can be strengthened through annual IAV vaccination of humans and support of sick leave policies for farm workers.

## Introduction

Influenza A virus (IAV) is one of the most important respiratory pathogens affecting animals and humans (1). Additionally, IAV infections occur worldwide and are considered endemic in swine populations (2). Influenza A virus in swine was the second most frequent confirmed disease etiology between 2010 and 2019 diagnosed from respiratory porcine tissue cases (3). Data from five Veterinary Diagnostic Laboratories (VDLs) in the U.S. showed an increase from 252 cases per month in 2009 to 2,244 cases tested per month in 2021 that were tested for IAV in swine (4). In addition, the USDA Influenza A Virus in Swine Surveillance program demonstrated an increase in the number of IAV submissions over time (https://www.aphis.usda.gov/animal_health/animal_dis_spec/swine/downloads/fy2017quarter2swinereport.pdf). Repeated outbreaks and the rapid spread of genetically and antigenically distinct IAV presents a considerable challenge for swine production (5). Due to the ability of IAV to infect swine and humans and rapidly evolve, there is a risk for zoonotic transmission from pigs to people, and the frequent incursions of human seasonal viruses into swine have greatly influenced the dynamics of IAV evolution (6).

The genetic diversity of IAV complicates efforts to control the virus, increasing the threat that a novel virus will emerge in pigs with the capacity to infect humans (7). Swine production systems in the United States (U.S.) rely on intensive farming systems comprising multiple sites to improve efficiency and profitability. Most farms are specialized in one of the stages of production, where pigs are transferred to a new location after the conclusion of a specific phase, which also presents a potential factor in the introduction and dissemination of infectious disease across farms and the U.S. (8). Implementation of animal health interventions are challenging because these require the initial financial investment of producers, and behavior changes to commit to and sustain these interventions over time (9).

Multiple factors influence animal health interventions chosen by veterinarians including economic costs, and production impacts. Swine veterinarians are on the front line of disease diagnosis and managing animal health decisions to control IAV, but limited information has been reported that evaluates their perception toward IAV infection in swine, implementation of control methods, or use of IAV vaccines. The objective of this study was to assess veterinarian perceptions of IAV prevention and control in U.S. swine populations.

## Materials and methods

A list of veterinarians was created from a client database provided by the Iowa State University Veterinary Diagnostic Laboratory (ISU-VDL) in 2017. The ISU-VDL receives porcine diagnostic submissions from all 50 states in the U.S. In addition, 75% of the ISU-VDL diagnostic submissions are swine for diagnosing disease or for surveillance of significant pathogens. An email invitation was sent to all veterinarians (*n* = 348) who had email addresses on April 21, 2017, associated with the ISU VDL. The initial survey email included a list of 22 questions (Supplementary material A) that could be answered through a link provided in the body of the email. Survey packets with a cover letter, hard copy of the survey document, and a postage-paid return envelope were mailed to 240 veterinarians who had not completed the online survey within 1 month or did not have a valid email address. The veterinarians were allowed 3 months to return the survey on-line or provide the hard copy through mail. The survey was sent to individual veterinarians and each participant had the opportunity to provide a response. Based on the American Association of Swine Veterinarian membership list, the 345 veterinarians contacted in the survey represented 82.53% (345/418) of the veterinarians working with swine in the U.S. The survey was approved by the Iowa State University Institutional Review Board (protocol number 17–027) and disseminated in cooperation with the Center for Survey Statistics and Methodology—Survey research services (CSSM-SRS) at Iowa State University.

The survey included questions related to: (a) Demographic variables: primary veterinary practice type, veterinarian age, geographic region of veterinary service (region 1 to 5 as per Table 1), the average size of breeding herds, and number of nursery and grow-finisher pigs served by the veterinarian. The response variable included the primary veterinary practice that had 3 levels: (1) swine exclusive veterinary practitioner, (2) large animal practitioner which corresponds to veterinarians that work predominantly with food animals such as bovine, porcine, ovine, caprine and poultry, and (3) mixed animal practitioner that corresponds to veterinarians working with companion and food animals. The predictor variables were (I) Veterinarian perceptions: relative importance of IAV, if IAV challenges are increasing or not; IAV economic impact; veterinarian and their client's (swine producers) perceived level of concern regarding IAV; and estimated cost per market hog of IAV in swine production systems; a need for a new or novel vaccine platform; and if the U.S. should continue to fund the USDA IAV swine surveillance program; (II) Control measures: percent of replacement gilt isolation, percent of breeding herds, and percent of nursery and grow-finisher sites using IAV vaccines; (III) Prevention measures: IAV vaccine platform used in breeding herds (autogenous or commercial vaccine), recommended time of vaccine administration in the breeding herd, use of whole-herd (mass) vaccination, primary source of IAV lateral transmission, farm worker annual IAV vaccine recommendations, suggested use of sick leave policy (sick leavy policy is a paid absence from duty, and an employee is entitled to use sick leave for personal or family medical needs), and use of personal protective equipment. The perceptions and opinions consider how veterinarians choose to monitor, control, and prevent IAV in swine, as a zoonotic concern. This also includes which control and prevention measures the veterinarians have been positioning within the primary veterinary practices (swine exclusive, large animal, and mixed animal practice).

**Table 1:** Influenza A virus veterinarian survey respondent demographic characteristics and description of veterinary practice types.

**Variable**	**Category**	**Veterinary practices**			**Frequency**	**Total (%)**
		**Swine^1^**	**Large^2^**	**Mixed3**		
Veterinarian age	≤ 30 years	28	6	1	35	18.6
	31–40 years	38	8	1	47	25.0
	41–50 years	22	7	2	31	16.5
	51–60 years	20	18	7	45	23.9
	>60 years	18	10	2	30	16.0
	Total	126	49	13	188	
Veterinarian practice region	Region 1	18	2	0	20	10.6
	Region 2	90	42	11	143	76.1
	Region 3	9	0	0	9	4.8
	Region 4	7	5	2	14	7.4
	Region 5	2	0	0	2	1.1
	Total	126	49	13	188
Average size of breeding herd (number of sows)	≤ 1,000	8	10	8	26	13.8
	1,001–5,000	81	33	3	117	62.2
	>5,001	33	6	1	40	21.3
	Unknown	4	0	1	5	2.7
	Total	126	49	13	188
Number of nursery and grow/finish pigs (total per year)	≤ 100,000 pigs	10	4	7	21	11.2
	100,001–500,000	31	27	4	62	33.0
	500,001–1,000,000	32	15	1	48	25.5
	More than 1,000,000	51	3	0	54	28.7
	Unknown	2	0	1	3	1.6
	Total	126	49	13	188

^1^Swine, represents swine exclusive veterinary practitioner; ^2^Large, represents large animal practitioner which corresponds to veterinarians that work predominantly with food animals; ^3^Mixed, represents mixed animal practitioner that corresponds to veterinarians working with companion and food animals.

Descriptive statistics were used to report the results from response variables. Fisher's exact test was performed to assess differences in proportion between veterinary practices for each question in the survey related to veterinarians' perceptions, control, and prevention measures against IAV. The significance was set at *P* ≤ 0.05, and a pairwise comparison was performed for the variables with *P* ≤ 0.05 to identify differences among the primary veterinary practice types (swine exclusive vs. large animal vs. mixed animal). All descriptive statistics and statistical analysis were performed using the R program v 4.1.0 (10).

## Results

**Figure 1:**
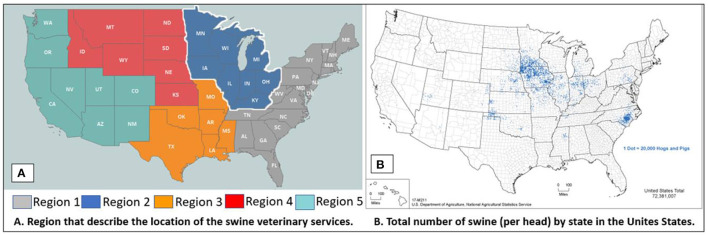
Regions represented by the influenza A virus veterinarian survey respondents **(A)** and relationship to the density of swine located in the United States **(B)**. *(B) Inventory of hogs and pigs from Census of Agriculture (11).

Complete surveys were received from 56.2% (194/345) veterinarians, with 68.0% (132/194) completed online and 31.9% (62/194) on paper. Three respondents were classified not eligible as they were not veterinarians actively engaged in the practice of veterinary medicine, five letters or survey packets were undeliverable, and two respondents completed a partial survey. In the present study, the veterinarians who classified themselves in the veterinary practice as “other” (*n* = 6) were not included in the analysis because these veterinarians were involved in alternative or unique practices, which precluded an accurate analysis. After eliminating extraneous respondents, the final response rate was 54.5% (188/345). Of the veterinarians that responded to the survey, 67% (126/188) reported swine exclusive, 26% (49/188) reported in the animal large category, and 7% (13/188) reported in the mixed animal category. Over one-third of the sampled veterinarians were from the state of Iowa, nearly one-half were from other U.S. Midwestern states, and the remainder were from across the U.S. (Figure 1). Demographics of the 188 survey respondents is described in Table 1.

The survey participants included veterinarians from 22 states in the U.S. Region 2 contained the highest number of respondents from the Midwestern U.S. which included Illinois, Indiana, Iowa, Kentucky, Michigan, Minnesota, Ohio, and Wisconsin representing 78.1% (147/188) of the responses. This region also includes the highest density of swine in the U.S. (Figure 1) (11).

Regarding the importance of IAV in the veterinarian's swine production systems they provide veterinary care, 89.4% (168/188) considered IAV one of the top three health challenges in the swine industry. The other two animal health issues of veterinarian concern include porcine reproductive and respiratory syndrome (PRRS) and porcine epidemic diarrhea (PED) (12, 13). In addition, 67.0% (126/188) of the veterinarians surveyed considered IAV challenges are increasing *(P* = 0.02), primarily observed between the responses from veterinarians practicing exclusively with swine (72.2%) and the mixed animal group (30.8%) with a significant difference (*P* = 0.01). Moreover, 66.0% (124/188) of the veterinarians considered IAV to have a moderate economic impact in swine, and there was a significant difference (*P* = 0.004) on the perception of the IAV economic impact among veterinary practices, swine exclusive and large animal veterinarians had different perceptions compared to mixed animal veterinarians (*P* = 0.002).

A total of 68.4% (128/187) veterinarians considered an estimated cost per market hog due to the presence of IAV in their clients' production systems between $1 and $5 per animal. Approximately 45.7% (86/188) veterinarians responded that swine producers representing their clients were somewhat concerned with IAV compared to those that reported higher levels or were unconcerned regarding IAV. However, 46.2% (86/186) of surveyed veterinarians reported they were concerned with IAV (*P* = 0.041) in swine, mostly among veterinarians responding as swine exclusive and large animal practitioners vs. mixed animal (*P* = 0.02). There were 74.2% (138/186) of veterinarians that responded the U.S. swine industry needs new or novel vaccine platforms (*P* = 0.001), with different opinions reported primarily between swine exclusive vs. mixed animal veterinarians (*P* < 0.001), and large animal vs. mixed animal veterinarians (*P* = 0.01). In addition, 80.6% (150/186) reported the U.S. should continue funding the federal USDA IAV surveillance program in swine that has been in operation since 2010 to monitor the presence of IAV strains in U.S. swine. The USDA influenza A virus surveillance in swine (https://www.aphis.usda.gov/animal_health/animal_dis_spec/swine/downloads/fy2017quarter2swinereport.pdf) is a federal program that was implemented after the 2009 H1N1 pandemic in humans that was caused by an IAV consisting entirely of swine lineage segments. The surveillance program monitors the genetic diversity of IAV in swine, detects new or emerging strains of IAV in pigs as well as the spillover of human IAV into swine. Ultimately, the surveillance program is designed to help track and monitor the emergence of potential pandemic IAV in humans that start in the swine population.

For control of IAV in breeding herds, most veterinarians reported use of vaccine in more than 50% of gilt development and breeding herds, 43.1% (81/188) of veterinarians indicated that 76–100% of their breeding herds used IAV vaccines, and 53.7% (101/188) reported that 76–100% of their gilt replacement or gilt development units used IAV vaccines. Currently, the IAV vaccines approved for use in swine are all inactivated and include commercial whole virus, autogenous or farm specific whole virus and replicon particle vaccines considered subunit vaccines (14, 15). In addition, only 34.0% (64/188) of the respondents reported that equal or <25% of their nursery and grow-finish sites use IAV vaccines. Of those herds using IAV vaccines, 84.8% (156/184) reported the use of commercial IAV vaccines and 50.0% (92/184) reported use of autogenous vaccines in the breeding herd, with a significant difference (*P* = 0.031) between responses from swine exclusive (55.3%) and mixed animal practitioners (15.4%). Vaccines are one of the main tools available to help control IAV, however, vaccination is often challenged by IAV genetic diversity due to mutations and re-assortment. Swine exclusive veterinarian responses differed in using autogenous vaccines compared to veterinarians in the other types of veterinary practices (Table 2). Unfortunately, DIVA vaccines (differentiating infected from vaccinated animals) are not currently available for use in the United States and the only live attenuated influenza virus (LAIV) vaccine available in the U.S was removed from commercial use in 2020.

**Table 2:** Survey responses regarding influenza A virus control and prevention strategies reported by veterinarians in swine exclusive, large animal, or mixed animal veterinary practices.

**Variable**	**Category**	**Veterinary practices**			**Total %**	*P*-value^*^
		**Swine %**	**Large %**	**Mixed %**		
Importance IAV in swine	Primary	2.3 (3/126)	0.0 (0/49)	0.0 (0/13)	1.6 (3/188)	0.079
	One of top three	87.3 (110/126)	98 (48/49)	76.9 (10/13)	89.4 (168/188)
	Minor	10.3 (13/126)	2.0 (1/49)	23.1 (3/13)	9.0 (17/188)
Perception of IAV health challenges	Increasing	72.2 (91/126)	63.3 (31/49)	30.8 (4/13)	67.0 (126/188)	0.020
	Stable	26.2 (33/126)	34.7 (17/49)	61.5 (8/13)	30.9 (58/188)
	Decreasing	0.0 (0/126)	0.0 (0/49)	0.0 (0/13)	0.0 (0/188)
	No opinion or unsure	1.6 (2/126)	2.0 (1/49)	7.7 (1/13)	2.1 (4/188)
Economic impact IAV in swine	High	27.8 (35/126)	28.6 (14/49)	0.0 (0/13)	26.1 (49/188)	0.004
	Moderate	65.1 (82/126)	69.4 (34/49)	61.5 (8/13)	66 (124/188)
	Low	6.3 (8/126)	2.0 (1/49)	30.8 (4/13)	6.9 (13/188)
	No opinion or unsure	0.8 (1/126)	0.0 (0/49)	7.7 (1/13)	1.1 (2/188)
Veterinarian IAV level concern	Very concerned	22.6 (28/124)	14.3 (7/49)	7.7 (1/13)	19.4 (36/186)	0.041
	Concerned	46.0 (57/124)	55.1 (27/49)	15.4 (2/13)	46.2 (86/186)
	Somewhat concerned	28.2 (35/124)	30.6 (15/49)	76.9 (10/13)	32.3 (60/186)
	Unconcerned	2.0 (3/124)	0.0 (0/49)	0.0 (0/13)	1.6 (3/186)
	No opinion or unsure	0.8 (1/124)	0.0 (0/49)	0.0 (0/13)	0.5 (1/186)
Client's IAV level concern	Very concerned	10.3 (13/126)	8.2 (4/49)	0.0 (0/13)	9 (17/188)	0.767
	Concerned	43.7 (55/126)	44.9 (22/49)	30.8 (4/13)	43.1 (81/188)
	Somewhat concerned	42.9 (54/126)	46.9 (23/49)	69.2 (9/13)	45.7 (86/188)
	Unconcerned	2.4 (3/126)	0.0 (0/49)	0.0 (0/13)	1.6 (3/188)
	No opinion or unsure	0.8 (1/126)	0.0 (0/49)	0.0 (0/13)	0.5 (1/188)
Estimated cost per hog ($)	≤ $1.00	14.4 (18/125)	8.2 (4/49)	15.4 (2/13)	12.8 (24/187)	0.422
	$1.01–$5.00	70.4 (88/125)	67.3 (33/49)	53.8 (7/13)	68.4 (128/187)
	$5.01–$10.00	7.2 (9/125)	10.2 (5/49)	15.4 (2/13)	8.6 (16/187)
	>$10.00	0.8 (1/125)	4.1 (2/49_	0.0 (0/13)	1.6 (3/187)
	Unknown	7.2 (9/125)	10.2 (5/49)	15.4 (2/13)	8.6 (16/187)
Does the US swine industry need new or novel vaccine platforms?	Yes	79.0 (98/124)	73.5 (36/49)	30.8 (4/13)	74.2 (138/186)	0.001
	No	0.8 (91/124)	2.0 (1/49)	0.0 (0/13)	1.1 (2/186)
	Depends on	16.1 (20/124)	16.3 (8/49)	30.8 (4/13)	17.2 (32/186)
	No opinion	4.0 (5/124)	8.2 (4/49)	38.5 (5/13)	7.5 (14/186)
Should the US continue funding an IAV surveillance program?	Yes	85.5 (106/124)	69.4 (34/49)	76.9 (10/13)	80.6 (150/186)	0.106
	No	4.8 (6/124)	12.2 (6/49)	0.0 (0/13)	6.5 (12/186)
	Only breeding herds	1.6 (2/124)	4.1 (2/49)	0.0 (0/13)	2.2 (4/186)
	Only nursery/finish	0.8 (1/124)	4.1 (2/49)	7.7 (1/13)	2.2 (4/186)
	Only sentinel by state	7.3 (9/124)	10.2 (5/49)	15.4 (2/13)	8.6 (16/186)

* *P*-value identifies the variables where at least two veterinary practices had a statistical difference on the proportion of responses. A second pairwise comparison was conducted if the *P*-value ≤ 0.05 to assess which groups differed from each other.

Of the responding veterinarians, 82.6% (152/184) suggested vaccination during gilt isolation, with a significant difference (*P* < 0.001) between responses from swine exclusive (76.4%) and large animal veterinarians (98%). For IAV vaccine use prior to farrowing, 57.4% (105/183) of the veterinarians suggested vaccination during this phase (*P* = 0.01), with a significant difference (*P* = 0.02) between responses from large animal (69.4%) and mixed animal practitioners (25%). In addition, only 26.5% (48/181) recommended quarterly vaccination, and only 38.3% (69/180) recommended biannual mass vaccination in the breeding herd. For the primary source of IAV introduction into swine populations, 53.8% (100/186) reported the neighboring pig farms were the likely source of lateral infection (Table 2).

Regarding swine farm employee IAV biosecurity practices in the workplace, 84.9% (158/186) of veterinarians recommend farm workers to receive an annual human influenza vaccine, and 51.6% (96/186) of veterinarians suggest the use of a sick leave policy to help control transmission of IAV between people and pigs (Table 2).

## Discussion

This study evaluated veterinarian perceptions and attitudes regarding IAV prevention, control, and biosecurity methods in swine in the U.S. Collectively, the respondents of this survey reported their perception of health challenges related to IAV is increasing in swine, IAV has a moderate economic impact, and veterinarians are concerned about the presence of IAV circulating in swine (Table 3). The veterinarians in swine exclusive practices may have different levels of concern for IAV compared to responses from veterinarians in large or mixed animal veterinary practices although this outcome was expected. This may be influenced by the fact that swine exclusive veterinarians work intensively with pigs on a routine basis, receive more swine-focused training, and are more involved with IAV challenges occurring on swine farms compared to others.

**Table 3:** Survey responses regarding the perceptions of influenza A virus reported by veterinarians in swine exclusive, large animal, or mixed animal veterinary practices.

**Variable**	**Category**	**Veterinary practices**			**Total %**	*P*-value^*^
		**Swine %**	**Large %**	**Mixed %**		
Use of IAV vaccines in gilt development	None	6.3 (8/126)	6.1 (3/49)	23.1 (3/13)	7.4 (14/188)	0.576
	≤ 25%	11.9 (15/126)	12.2 (6/49)	15.4 (2/13)	12.2 (23/188)
	26–50%	5.6 (7/126)	10.2 (5/49)	7.7 (1/13)	6.9 (13/188)
	51–75%	16.7 (21/126)	20.4 (10/49)	15.4 (2/13)	17.6 (33/188)
	76–100%	56.3 (71/126)	51.0 25/49)	38.5 (5/13)	53.7 (101/188)
	Unknown	3.2 (4/126)	0.0 (0/49)	0.0 (0/13)	2.1 (4/188)
Use of IAV vaccines in breeding herds	None	6.3 (8/126)	4.1 (2/49)	23.1 (3/13)	6.9 (13/188)	0.276
	≤ 25%	15.9 (20/126)	14.3 (7/49)	15.4 (2/13)	15.4 (29/188)
	26–50%	12.7 (16/126)	8.2 (4/49)	7.7 (1/13)	11.2 (21/188)
	51–75%	19.0 (24/126)	24.5 12/49)	38.5 (5/13)	21.8 (41/188)
	76–100%	43.7 (55/126)	49.0 24/49)	15.4 2/13)	43.1 (81/188)
	Unknown	2.4 (3/126)	0.0 (0/49)	0.0 (0/13)	1.6 (3/188)
Use of IAV vaccines in nursery and grow finish swine	None	61.9 (78/126)	44.9 (22/49)	38.5 (5/13)	55.9 (105/188)	0.389
	≤ 25%	27.8 (35/126)	44.9 (22/49)	53.8 (7/13)	34.0 (64/188)
	26–50%	4.8 (6/126)	4.1 (2/49)	7.7 (1/13)	4.8 (9/188)
	51–75%	1.6 (2/126)	2.0 (1/49)	0 (0/13)	1.6 (3/188)
	76–100%	2.4 (3/126)	2.0 (1/49)	0 (0/13)	2.1 (4/188)
	Unknown	1.6 (2/126)	2.0 (1/49)	0 (0/13)	1.6 (3/188)
Use of commercial vaccines in breeding herds	Yes	83.7 (103/123)	87.5 (42/48)	84.6 (11/13)	84.8 (156/184)	0.689
	No	13.8 (17/123)	10.4 (5/48)	7.7 (1/13)	12.5 (23/184)
	Do not know	2.4 (3/123)	2.1 (1/48)	7.7 (1/13)	2.7 (5/184)
Use of autogenous vaccines in breeding herds	Yes	55.3 (68/123)	45.8 (22/48)	15.4 (2/13)	50.0 (92/184)	0.038
	No	42.3 (52/123)	50.0 (24/48)	76.9 (10/13)	46.7 (86/184)
	Do not know	2.4 (3/123)	4.2 (2/48)	7.7 (1/13)	3.3 (6/184)
Recommend IAV vaccines in gilt isolation	Yes	76.4 (94/123)	98.0 (48/49)	83.3 (10/12)	82.6 (152/184)	0.0006
	No	23.6 (29/123)	2.0 (1/49)	16.7 (2/12)	17.4 (32/184)
Recommend IAV vaccines pre-breeding	Yes	26.8 (33/123)	41.7 (20/48)	16.7 (2/12)	30.1 (55/183)	0.110
	No	73.2 (90/123)	58.3 (28/48)	83.3 (10/12)	69.9 (128/183)
Recommend IAV vaccines pre-farrowing	Yes	55.7 (68/122)	69.4 (34/49)	25.0 (3/12)	57.4 (105/183)	0.017
	No	44.3 (54/122)	30.6 (15/49)	75.0 (9/12)	42.6 (78/183)
Quarterly IAV mass vaccination	Yes	25.6 (31/121)	26.5 (13/49)	36.4 (4/11)	26.5 (48/181)	0.715
	No	74.4 (90/121)	73.5 (36/49)	63.6 (7/11)	73.5 (133/181)
Biannual IAV mass vaccination	Yes	37.5 (45/120)	38.8 (19/49)	45.5 (5/11)	38.3 (69/180)	0.900
	No	62.5 (75/120)	61.2 (30/49)	54.5 (6/11)	61.7 (11/180)
Primary source of IAV lateral transmission	Replacement gilts	35.5 (44/124)	22.4 (11/49)	23.1 (3/13)	31.2 (58/186)	0.185
	Regional pig farm	48.4 (60/124)	67.3 (33/49)	53.8 (7/13)	53.8 (100/186)
	Humans	14.5 (18/124)	8.2 (4/49)	15.4 (2/13)	12.9 (24/186)
	Other source	1.6 (2/124)	2.0 (1/49)	7.7 (1/13)	2.2 (4/186)
Recommend workers annual IAV vaccine	Yes	87.9 (109/124)	77.6 (38/49)	84.6 (11/13)	84.9 (158/186)	0.186
	No	9.7 (12/124)	20.4 (10/49)	7.7 (1/13)	12.4 (23/186)
	No opinion	2.4 (3/124)	2.0 (1/49)	7.7 (1/13)	2.7 (5/186)
Sick leave policy	Yes	47.6 (59/124)	59.2 (29/49)	61.5 (8/13)	51.6 (96/186)	0.374
	No	17.7 (22/124)	14.3 (7/49)	0.0 (0/13)	15.6 (29/186)
	Depends on severity	28.2 (35/124)	18.4 (9/49)	38.5 (5/13)	26.3 (49/186)
	No opinion	6.5 (8/124)	8.2 (4/49)	0.0 (0/13)	6.5 (12/186)
Recommend use of coveralls/Tyvek	Yes	91.9 (114/124)	93.9 (46/49)	76.9 (10/13)	91.4 (170/186)	0.163
	No	8.1 (10/124)	6.1 (3/49)	23.1 (3/13)	8.6 (16/186)
Recommend use of gloves	Yes	81.5 (101/124)	73.5 (36/49)	69.2 (9/13)	78.5 (146/186)	0.321
	No	18.5 (23/124)	26.5 (13/49)	30.8 (4/13)	21.5 (40/186)
Recommend use of respirator/mask (N95)	Yes	45.2 (56/124)	59.2 (29/49)	53.8 (7/13)	49.5 (92/186)	0.222
	No	54.8 (68/124)	40.8 (20/49)	46.2 (6/13)	50.5 (94/186)
Recommend use of boots	Yes	97.6 (121/124)	98.0 (48/49)	100 (13/13)	97.8 (182/186)	0.990
	No	2.4 (3/124)	2.0 (1/49)	0.0 (0/13)	2.2 (4/186)

* *P*-value identify the variables where at least two veterinary practices had a statistical difference on the proportion of responses. A second pairwise comparison was conducted if the *P*-value ≤ 0.05 to assess which groups differed from each other.

Veterinarians are often considered the main source of information concerning disease prevention and biosecurity, and it is of utmost importance that these veterinarians be familiar with current information on disease prevention, control, and biosecurity methods and that they have the ability to communicate this in the best possible format to swine producers and farmers (16). Furthermore, veterinarians are expected to understand all processes and procedures associated with swine production beyond diagnosing disease. This includes broad knowledge related to management, nutrition, and economic decisions. Thus, due to the consistent increasing need to prevent and control important diseases such as IAV, there is a demand that veterinarians be familiar with all aspects of swine production. Therefore, differences in veterinary practice types may have influenced the responses that are reported in this survey based on the significant differences often observed between swine exclusive and large or mixed animal veterinarians.

The responses of this survey may suggest swine exclusive veterinarians are perhaps more aware of the complex genetic diversity related to IAV and the need to have farm-specific vaccines for adequate control. In addition, the majority of veterinarians responding to the survey reported the U.S. swine industry needs new or novel vaccine platforms likely due to the current IAV challenges faced in the field and the need for alternative vaccine options to improve cross-protection against different strains of the virus (17).

These results also suggest veterinarians consider it important to recommend an annual IAV vaccination for farm employees, and a sick leave policy to help protect against bi-directional or inter-species transmission of the virus. A recent study demonstrated the risk of IAV transmission between farm workers and pigs through detection of a swine-lineage IAV in the nasal passage of an employee, which emphasizes the need to implement biosecurity practices or protocols at the pig and human interface (18). Moreover, veterinarians play an important role in promoting public health by educating clients about zoonotic diseases such as IAV (19). The interface between swine farm workers and pigs represents an opportunity for veterinarians to promote awareness of zoonotic diseases and associated risks involving IAV in swine farms and implement appropriate biosecurity practices.

The current IAV swine vaccine platforms are based on whole inactivated virus (WIV), which could be commercial or autogenous vaccines, vectored or RNA vaccines, and live attenuated influenza vaccines (LAIV). Unfortunately, the NS1-truncated LAIV has not been commercially available since 2020 due to re-assortment with endemic wild-type strains in the U.S. swine population (20). The efficacy of current vaccines would be greater if IAV strains were updated more frequently to improve the likelihood of antibodies matching the prevalent circulating strains (15). Additionally, an integrated multi-agency approach is needed to improve IAV vaccine strain selection for use in swine (17).

Multiple pandemic H1N1 spillovers from humans to swine have occurred since 2009, and human-like H1 (pandemic clade) viruses have become one of the major lineages of IAV detected and characterized from swine respiratory disease (21–23). Thus, there is a need to prevent bidirectional IAV transmission between pigs and humans. Implementing and strengthening more human preventive strategies such as human IAV vaccination, sick-leave policies, and the use of personal protective equipment by farm workers is essential in the prevention against IAV, benefiting swine and human health.

This survey has potential limitations due to the risk of selection bias that may occur due to the non-response rate of the study population, which occurs when responses are potentially different between those involved in the survey compared to those who did not respond (24). Another potential limitation is related to the potential type I error given that multiple pairwise comparisons were performed across different levels of the outcome variable. However, the results of this study can be applied to the U.S. swine population when considering the internal validity of the responses, particularly in regions with the highest response rate. Nonetheless, for the external validity, the results from this survey may have different perceptions and attitudes related to IAV prevention, control, and biosecurity methods due to regional and cultural aspects. The results should be interpreted with caution in regions with fewer pigs or other countries where swine production or level of IAV circulation is much different compared to the U.S.

Although this survey occurred in 2017, the data from this survey is valuable to the U.S. swine industry. Veterinarians have yet to be approached with basic questions regarding their impressions of IAV that remain applicable regardless of when the survey occurred; thus, data from this study still reflects what veterinarians have been challenged with in field conditions. Moreover, the genetic variability of IAV in swine is constantly changing; however, the methods and tools available for surveillance and control, including vaccine platforms used in swine, have stayed the same, and veterinarians remain frustrated with the inability to control IAV.

The associated differences in the responses among the different types of veterinary practices emphasize the importance of veterinarians' concerns regarding IAV in the U.S. swine population (Table 3). The different responses between veterinarians in swine exclusive practices may be influenced by their level of experience, knowledge of swine diseases, and integral connection with the swine industry although this requires a more thorough investigation. In addition, this survey highlighted the opportunity to increase or improve biosecurity recommendations for swine farm employees such as receiving the human IAV vaccine annually and the use of respiratory protection, and to implement use of a sick leave policy if vaccines and/or vaccination is not an option, all of which can help reduce bi-directional transmission of the virus.

This study assessed veterinarian perceptions regarding IAV in swine production systems and control and prevention methods from the breeding herd through grow-finisher stages of production. This study also presented different perceptions regarding IAV among veterinarians in different types of veterinary practices. It described the current IAV mitigation practices implemented in swine farms based on strategic decisions. This study also revealed the veterinarians' perceptions that IAV as a health problem in swine is increasing, IAV has a moderate economic impact, and there is a high level of concern regarding IAV circulating in swine. These findings highlight the need for IAV surveillance data, improved vaccine platforms and strategies, as well as important opportunities regarding methods of control and biosecurity. Additionally, biosecurity practices associated with the veterinarian's swine operations and prevention of zoonotic diseases can be strengthened through IAV annual human vaccination and support of sick leave policies for farm workers.

## Data availability statement

The original contributions presented in the study are included in the article/Supplementary material, further inquiries can be directed to the corresponding author.

## Ethics statement

The studies involving human participants were reviewed and approved by the Iowa State University Institutional Review Board (protocol number 17-027) and disseminated in cooperation with the Center for Survey Statistics and Methodology—Survey Research Services (CSSM-SRS) at Iowa State University. The patients/participants provided their written informed consent to participate in this study.

## Author contributions

PG, AV, and JZ designed the study. PG, XW, ZZ, and EB coordinated the survey and data collection. DM, GS, DL, and PG did the formal analyses. SJ designed Figure 1A. DM lead the manuscript writing. All authors contributed to writing and revision and approved the final manuscript.
