# Supplementary material for: Veterinarian perceptions and practices in prevention and control of influenza virus in the Midwest United States swine farms
Source: Front Vet Sci. 2023 Feb 3;10:1089132. doi: 10.3389/fvets.2023.1089132 (PMC9936088; doi:10.3389/fvets.2023.1089132)
Supplement: Supplementary file 1 [file Table_1.DOCX]

Supplementary Material

## Supplementary Material A

**Supplemental Information**

***Survey of Veterinarians: Influenza A Virus (IAV) in U.S. Swine***

***For each question, please circle the response that most accurately reflects your experiences and opinions.***

**General Questions.**

1. What is your primary veterinary practice/specialty?

1 = Swine exclusive

2 = Large animal exclusive

3 = Mixed animal

4 = Mixed animal-predominantly large

5 = Other *(Please specify: ________________________________________________ )*

1. What is your age category?

1 = ≤ 30

2 = 31-40

3 = 41-50

4 = 51-60

5 = ≥ 61

1. Which geographic region best describes the location of your swine veterinary services?

1 = Region 1: AL, CT, DE, FL, GA, ME, MD, MA, NH, NJ, NY, NC, PA, RI, SC, TN, VT, VA, WV

2 = Region 2: IL, IN, IA, KY, MI, MN, OH, WI

3 = Region 3: AR, LA, MS, MO, OK, TX

4 = Region 4: ID, KS, MT, NE, ND, SD, WY

5 = Region 5: AK, AZ, CA, CL, HI, NV, NM, OR, UT, WA

1. What is the average size of the breeding herds (# of sows) you currently provide with veterinary services?

1 = ≤ 1,000 sows

2 = 1,001-5,000 sows

3 = 5,001-25,000 sows

4 = More than 25,000 sows

5 = Unknown/Not Applicable

1. How many nursery/grow/finish pigs (total per year) do you currently provide with veterinary services or influence in one year?

= ≤ 100,000 pigs

2 = 100,001-500,000 pigs

3 = 500,001-1,000,000 pigs

4 = More than 1,000,000 pigs

5 = Unknown/Not Applicable

1. Where would you say IAV ranks among the major health challenges in the US swine industry?

1 = The primary health challenge in the swine industry

2 = One of the top three health challenges in the swine industry

3 = Minor health challenge in the swine industry

4 = Not a health challenge in the swine industry

1. Do you perceive IAV to be an increasing, stable or decreasing health problem in swine?

1 = Increasing

2 = Stable

3 = Decreasing

4 = No opinion, Unsure

1. What would you say is the level of economic impact regarding the presence of IAV in swine?

1 = High economic impact

2 = Moderate economic impact

3 = Low economic impact

4 = No economic impact

5 = No opinion, Unsure

1. What is your clients’ level of concern regarding the presence of IAV in their swine operations?

1 = Very Concerned

2 = Concerned

3 = Somewhat Concerned

4 = Unconcerned

5 = No opinion, Unsure

1. What would you estimate is the cost per market hog of IAV in your clients’ swine operations, based on production losses and treatment costs due to infection or clinical disease?

1 = ≤ $1.00

2 = $1.01 - $5.00

3 = $5.01 - $10.00

4 = More than $10.00

5 = Unknown/Not Applicable

**Influenza Prevention & Control.**

1. Among your clients, what percent of **breeding/gestation farms** use IAV vaccines?

1 = None

2 = ≤ 25%

3 = 26-50%

4 = 51-75%

5 = 76-100%

6 = Unknown

1. Among your clients, what percent of **GDU/replacement gilt isolation** use IAV vaccines?

1 = None

2 = ≤ 25%

3 = 26-50%

4 = 51-75%

5 = 76-100%

6 = Unknown

1. Among your clients, what percent of **nursery/grow/finish sites/farms** use IAV vaccines?

1 = None

2 = ≤ 25%

3 = 26-50%

4 = 51-75%

5 = 76-100%

6 = Unknown

1. What type(s) of IAV vaccines are used in your clients’ breeding/gestation farms?

|  |  | Yes | No | Don’t Know |
| --- | --- | --- | --- | --- |
| a. | Commercial vaccine | 1 | 2 | 3 |
| b. | Autogenous vaccine | 1 | 2 | 3 |
| c. | Replicon particle (RP) subunit vaccine (Merck Animal Health) | 1 | 2 | 3 |
| d. | Other IAV type (Specify: _____________________) | 1 | 2 | 3 |

1. When do you suggest your clients use an IAV vaccine in the breeding herd?

|  |  | Yes | No |
| --- | --- | --- | --- |
| a. | During gilt isolation | 1 | 2 |
| b. | Pre-breeding | 1 | 2 |
| c. | Pre-farrowing | 1 | 2 |
| d. | Quarterly mass vaccination | 1 | 2 |
| e. | Biannual mass vaccination | 1 | 2 |

1. Which do you believe to be the primary source for introduction of new IAV into
   breeding/gestation farms?

1 = Replacement gilts

2 = Regional swine farms/nearby swine

3 = Humans

4 = Other source *(Please specify: _______________________________________)*

1. Does the US swine industry need new or novel vaccine platforms to help control IAV in swine?

1 = Yes

2 = No

3 = Depends

4 = No opinion

**Swine and Human Biosecurity.**

1. What is your level of concern regarding the presence of IAV in your clients’ swine operations?

1 = Very Concerned

2 = Concerned

3 = Somewhat Concerned

4 = Unconcerned

5 = No opinion, Unsure

1. Do you recommend that farm personnel use any of the following personal protective equipment
   when working with swine?

|  |  | Yes | No |
| --- | --- | --- | --- |
| a. | Coveralls/Tyvek | 1 | 2 |
| b. | Gloves | 1 | 2 |
| c. | Respirator/dust mask (N95) | 1 | 2 |
| d. | Boots | 1 | 2 |
| e. | Anything else? (Specify: _________________ ) | 1 | 2 |

1. Do you recommend that farm employees receive an annual human influenza vaccine?

1 = Yes

2 = No

3 = Recommended but not enforced

4 = No opinion, Not Applicable

1. Do you suggest your swine operations use a sick-leave policy for farm employees or visitors?

1 = Yes

2 = No

3 = Depends on the severity of influenza-like illness

(100.5°F fever, combination of 2 or more symptoms, etc.)

4 = No opinion, Not Applicable

1. Should the United States continue to fund an IAV surveillance program in swine?

1 = Yes

2 = No

3 = Only in breeding swine

4 = Only in nursery/grow/finish swine

5 = Only in a sentinel subset of herds in each state

1. Please record any comments you would like to make regarding IAV in swine in the U.S.

***Thank you very much for your assistance. Please return the completed survey in the envelope provided.***
